# Decoding motor intent from simulated multiple longitudinal intrafascicular electrode recordings

**DOI:** 10.1186/1471-2202-14-S1-P201

**Published:** 2013-07-08

**Authors:** Mohamed Abdelghani, James Abbas, Kenneth Horch, Ranu Jung

**Affiliations:** 1 Department of Biomedical Engineering, Florida International University, Miami, FL, 33174, USA; 2 School of Biological and Health Systems Engineering, Arizona State University, Tempe, AZ, 85287, USA

## 

**Figure 1:**
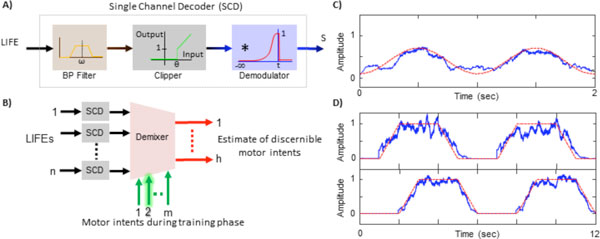
(A) LIFE single channel decoder (SCD): neural signals are bandpass filtered, clipped to remove any residual background noise and normalize spike amplitudes, and filtered to smooth the spike trains and obtain the modulating signal S (i.e. motor intent). (B) Multiple single channel decoders followed by a demixer. (C) Actual motor intent (Red) and decoded motor intent (Blue) for an input signal with a single motor intent. (D) Demixing of two overlapped motor intents; (Red) actual motor intents, (Blue) estimated motor intent signals after demixing.

Signals recorded from peripheral nerves may provide an effective and reliable means of controlling powered prosthetic limbs. Longitudinal intrafascicular electrodes (LIFEs) have been used to record extracellular motor activity from peripheral nerves in upper-limb amputees for periods up to several weeks and the ability to decode the activity and use it for single degree-of-freedom (DOF) control of a prosthetic arm has been demonstrated [1]. However, simultaneous control of multiple DOFs of the prosthesis, which is important for many daily tasks, presents additional challenges. Recently we developed a platform to simulate recording of extracellular motor activity from multiple LIFE electrodes [2]. We have also designed and tested an online decoding algorithm that utilizes these simulated recordings. Figure 1A&B shows the schematic of the decoder structure. The decoder is composed of multiple single channel decoders (SCDs) and a demixer. The SCD decodes motor intent from a LIFE recording. It is composed of a bandpass filter to attenuate noise and sharpen spikes, a clipping function to identify spikes and a half-Gaussian smoothing kernel to get a smoothed real-time estimate of motor intent. The demixer identifies the motor intent signals as corresponding to a particular motion class, such as wrist flexion, supination etc. The demixer requires a learning stage, where recordings from LIFEs are correlated to motion classes. A simple batched LMS algorithm is used to train the parameters of the demixer. Figure 1C, display the result of a single channel decoding of a sinusoidal motor intent. Figure 1D shows results of demixing of two overlapped motor intents recorded by two LIFEs: the first electrode records activity from the two motor pools while the second electrode records activity from only one (not shown). During the learning stage, the demixer learns to account for common motor intent and provide good estimates of the two different motor intent signals. The decoder developed here could be readily implemented in a real-time, portable, low-power configuration to translate multiple LIFE recordings to motor intent signals that enable multi-DOF control of a powered prosthesis.
